# Green Synthesis of Silver Nanoparticles Using *Spilanthes acmella* Leaf Extract and its Antioxidant-Mediated Ameliorative Activity against Doxorubicin-Induced Toxicity in Dalton’s Lymphoma Ascites (DLA)-Bearing Mice

**DOI:** 10.1021/acsomega.2c05970

**Published:** 2022-11-25

**Authors:** Fanai Lalsangpuii, Samuel Lalthazuala Rokhum, Fanai Nghakliana, Lal Fakawmi, Joseph V. L. Ruatpuia, Esther Laltlanmawii, Ralte Lalfakzuala, Zothan Siama

**Affiliations:** † Department of Botany, Mizoram University, Aizawl796004, Mizoram, India; ‡ Department of Chemistry, National Institute of Technology Silchar, Silchar788010, Assam, India; § Department of Zoology, Mizoram University, Aizawl796004, Mizoram, India

## Abstract

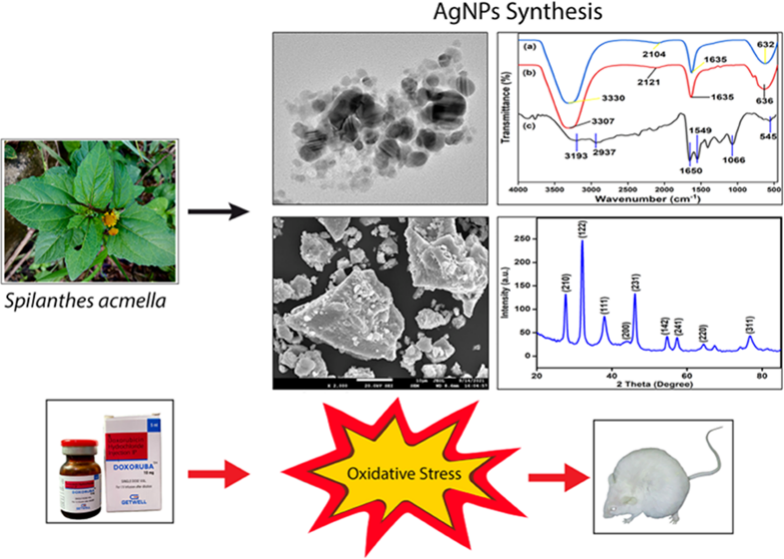

Green synthesis of metal nanoparticles is a rapidly growing research area in the field of nanotechnology because of their biomedical applications. This study describes the synthesis of silver nanoparticles (AgNPs) using *Spilanthes acmella* leaf extract and its ameliorative effects against doxorubicin-induced toxicity. The formation of AgNPs was confirmed by a ultraviolet–visible (UV–vis) spectrum that revealed an absorption band at 430 nm. A shift in the absorption bands in Fourier-transform infrared spectroscopy (FT-IR) confirmed the bioactive molecules of *S. acmella* leaf extract that acted as a reducing and capping agent. The spherical shape of AgNPs was confirmed by scanning electron microscope (SEM) analysis, and the presence of elemental silver was indicated by energy dispersive X-ray spectroscopy (EDS) analysis. X-ray diffraction (XRD) analysis revealed that the crystalline size of the synthesized AgNPs was 6.702 nm. Treatment of Dalton’s lymphoma ascites (DLA) mice with 20 mg/kg of doxorubicin (DOX) significantly increased the activities of serum toxicity markers including aspartate amino-transferase (AST), alanine amino-transferase (ALT), and lactate dehydrogenase (LDH). However, compared to DOX alone treatment, the coadministration of DOX and AgNPs reduced AST, ALT, and LDH activities. DOX alone treatment reduced glutathione (GSH) contents and decreased the activities of glutathione-s-transferase (GST) and superoxide dismutase (SOD) in DLA mice. However, the administration of AgNPs to DOX-treated DLA mice increased GSH content and the activities of GST and SOD. Consistently, biosynthesized AgNPs were found to possess significantly higher free-radical scavenging activities when compared to the *S. acmella* leaf extract, as measured by ABTS, DPPH, and O_2_^•**–**^ assays. The biosynthesized AgNPs also showed significant inhibitory activities against erythrocyte hemolysis and lipid peroxidation in the liver homogenate.

## 1 Introduction

Nanotechnology associated with metal nanoparticles emerges as a rapidly growing field in the realm of science and technology, principally in biomedical sciences due to its unique optical catalytic, electronic, magnetic, and thermal characteristics.^1^ The unique physicochemical characteristics of metal nanoparticles make them ideal for many biological applications, mostly due to the high surface-to-volume ratio.^2^ Metallic nanoparticles have several biomedical applications including antioxidant, antimicrobial, anticancer, anticoagulant, antidiabetic, and thrombolytic activities.^3−6^ Synthesis of nanoparticles can be carried out from different techniques broadly through physical, chemical, and biological methods. Biological methods for synthesizing metallic nanoparticles have received considerable attention because they utilize biological resources such as microorganisms, animal metabolites, marine algae, microfluids, and plant extracts to reduce and stabilize nanoparticles.^7−9^ Synthesis of silver nanoparticles (AgNPs) using plants provides an eco-friendly and adequate approach because plants are widely distributed, easily available, and devoid of the use of many expensive, toxic, and harmful chemical compounds.^10^ The synthesis of AgNPs follows a two-step process involving the reduction of Ag^+^ ions to Ag^0^ followed by the agglomeration and stabilization that lead to the formation of colloidal AgNPs.^11^ Biomolecules such as amino acids, proteins, NAD(P)+ reductases, dehydrogenases, and various secondary metabolites present in the plant extract reduced silver ions in the synthesis of AgNPs.^12^ Extracellular proteins, enzymes, or peptides formed the capping agents, which are absorbed onto the surface of AgNPs.^13^ In the synthesis of AgNPs, plant extract acts as both a reducing and stabilizing agent that protects from agglomeration and affects the morphology of nanoparticles by preventing their uncontrolled growth.^14^ The biosynthesis of AgNPs mediated by phytocompounds found in the plant extract can operate as efficient antioxidants or free-radical scavengers by reducing reactive oxygen species (ROS) generation and protecting various biomolecules.^15,16^ Thus, plant-based green synthesis platforms are endorsed as one of the best routes for producing metal nanoparticles.^17^

Doxorubicin (DOX) is an indispensable anthracycline antibiotic that displays a broad spectrum of anticancer activity and has been clinically used to treat various malignant neoplasms.^18^ Despite having an effective therapeutic role, DOX administration has been constrained due to cumulative dose-dependent effects that led to organ toxicity such as cardiotoxicity, hepatotoxicity, and nephrotoxicity, which subsequently reduces its clinical utility.^19^ Although the mechanisms involved in the onset of DOX-induced organ toxicity remain obscure, numerous studies have revealed it to be multifactorial, among which the induction of oxidative stress due to the generation of free radicals seems to be the key player. Free radicals are atoms, molecules, or ions with unpaired electrons which are biologically derived from oxygen, nitrogen, and sulfur molecules. When present in low to moderate concentrations, free radicals such as superoxide (O_2_^•**–**^), hydroxyl radicals (^•^OH), and singlet oxygen (^1^O_2_) are essential in regulating various physiological functions of the body.^20^ However, owing to their unpaired electron, they are extremely reactive with other cellular molecules and can hamper the body’s antioxidant defense systems, thereby leading to oxidative stress.^21^ DOX promotes oxidative stress by the formation of a semiquinone derivative *via* an NADPH-dependent reduction reaction. The redox cycling of semiquinone to quinone in the presence of oxygen generates superoxide radicals (O_2_^•**–**^). The superoxide radical produces several free radicals through a subsequent chain reaction, including hydrogen peroxide (H_2_O_2_) and hydroxyl ions (^•^OH).^22^ In addition, DOX generates free radicals *via* an iron ion-dependent nonenzymatic mechanism, thereby resulting in lipid peroxidation, DNA/RNA damage, inhibition of autophagy, disturbance of calcium homeostasis, and the subsequent activation of inflammatory response and apoptosis.^23^ Even though cells are equipped with a strong endogenous antioxidant system to counterbalance the increasing levels of ROS to prevent oxidative stress, it has been reported that DOX can suppress the endogenous antioxidant system such as glutathione and catalase, thereby promoting the accumulation of free radicals and subsequently causing redox imbalance.^24^ Thus, one strategy to combat DOX-induced organ toxicity is an exogenous supply of antioxidants.

Bioactive compounds isolated from medicinal plants have been reported to prevent DOX-induced toxicity due to their free-radical scavenging activities.^25,26^ In this study, we explored the ability of biosynthesized AgNPs using *Spilanthes acmella* leaf extract to provide protective actions against DOX-induced toxicity. *S. acmella*, commonly known as the toothache plant, is a member of the Asteraceae family. It is native to Brazil and widely distributed in different parts of the world including America, Australia, Africa, and India.^27^ This herd has been traditionally used for the treatment of various illnesses such as toothache, throat and gum infections, stomatitis, articular rheumatism, tuberculosis, and leucorrhea by different communities.^28^ Different parts of *S. acmella* have been shown to contain different bioactive groups including phenolics, alkyl amide, glycosides, coumarins, triterpenoids, and pyroglutamate with potent anesthetic, antipyretic, analgesic, antifungal, antimalarial, aphrodisiac, vasorelaxant, and immunomodulatory properties.^28,29^ Since increased ROS levels are frequently associated with DOX-induced toxicity, an approach to achieving redox homeostasis may represent an effective tactic to improve the therapeutic efficacy of doxorubicin. Thus, this study investigated the protective effects of biosynthesized AgNPs using *S. acmella* leaf extract against DOX-induced cardiotoxicity and hepatoxicity in Dalton′s lymphoma ascites (DLA)-bearing mice.

## 2 Methods

### 2.1 Chemicals and Reagents

Bovine serum albumin (BSA), 1,1-diphenyl-2-picrylhydrazyl radicals (DPPH), 2,2′-azino-bis-(3-ethylbenzothiazoline-6-sulfonic acid) (ABTS), nicotinamide adenine dinucleotide (NADH), nitroblue tetrazolium (NBT), disodium hydrogen phosphate, *n*-butyl alcohol, 2-thiobarbituric acid (TBA), phenazine methosulfate (PMS), glutathione reduced, potassium persulfate, gallic acid, quercetin dihydrate, ferric chloride, sodium nitrite, methanol, and cumene hydroperoxide were obtained from HiMedia Laboratories Pvt., Ltd. (Mumbai, India). Glacial acetic acid, aluminum chloride, silver nitrate, hydrogen peroxide (H_2_O_2_), and 5,5′-dithio-2-nitrobenzoic acid (DTNB) were obtained from Merck Specialities Pvt., Ltd. (Mumbai, India). Potassium ferricyanide and ferrous chlorides were obtained from Loba Chemie Pvt., Ltd. (Mumbai, India). 1-Chloro-2,4-dinitrobenzene (CDNB), cupric sulfate, trichloroacetic acid (TCA), Folin–Ciocalteu’s reagent, sodium hydroxide, and ascorbic acid were obtained from SD Finechem Ltd. (Mumbai, India). Doxorubicin (Getwell Oncology Pvt., Ltd., Haryana, India) was purchased from a local pharmacy.

### 2.2 Preparation of Plant Extract

Fresh leaves of *S. acmella* were collected from Aizawl, Mizoram, India. The leaves were washed, dried, and minced. Pulverized leaves (25 g) were boiled in distilled water (100 mL) for 1 h. The liquid extract was then centrifuged and filtered using Whatman No. 1 filter paper. After filtration, the extract was stored at 4 °C until further processed for the synthesis of AgNPs.

### 2.3 Preparation of 1 mM Silver Nitrate Solution

For the preparation of 1 mM silver nitrate (AgNO_3_), 17 mg of AgNO_3_ was dissolved in 100 mL of double distilled water. The solution was stored in an amber bottle to avoid light-induced oxidation of silver.

### 2.4 Synthesis of Silver Nanoparticles (AgNPs)

*S. acmella* leaf extract (10 mL) was mixed with 90 mL of 1 mM AgNO_3_ solution, and the pH of the mixture was maintained at 7.0. After stirring at 3 h at room temperature, the reaction mixture changed to reddish brown from light yellowish, indicating the synthesis of AgNPs. The biosynthesized AgNPs were centrifuged at 15,000 rpm for 5 min and redispersed in deionized water to eliminate any uncoordinated biological molecules. Further, the formation of AgNPs by the reduction of Ag^+^ from AgNO_3_ was ascertained by ultraviolet–visible (UV–vis) spectral analysis.

### 2.5 Characterization of Silver Nanoparticles

The formation of AgNPs in the colloidal solution was confirmed by the UV–vis spectrum (EI 2375 double beam UV–vis spectrophotometer) at 370–700 nm. The possible functional groups involved in the synthesis of AgNPs were investigated using Fourier-transform infrared spectroscopy (FT-IR) spectrum analysis (Perkin-Elmer Spectrum Two with Universal ATR Software 10; Spectrum 10.5.2.636). The crystalline metallic AgNPs were examined by X-ray diffraction analysis (X-ray diffractometer D8 ADVANCE ECO BRUKER) with a Cu Kα radiation (λ = 1.54060 Å, 40 kV, 30 mA) monochromatic filter in the range of 10–80° at 2θ angles. The morphology of biosynthesized AgNPs was investigated by transmission electron microscopy (TEM) (JEM-2100 Plus Electron Microscope, JEOL Ltd.) and scanning electron microscope (JSM-IT800 Schottky Field Emission; JEOL Ltd.).

### 2.6 Determination of Free-Radical Scavenging Activity (*In Vitro*)

#### 2.6.1 DPPH Radical Scavenging Activity

DPPH radical scavenging activity of the green synthesized AgNPs was assessed using the method describe earlier^30^ with minor modifications. Briefly, to different concentrations of AgNPs (0.5 mL, 1–10 μg/mL), 1 mL of methanol solution of DPPH (0.1 M) was added. After 30 min of incubation in the dark, the absorbance of the solution was measured at 523 nm using UV–visible spectrophotometer (SW 3.5.1.0. Biospectrometer, Eppendorf India Ltd., Chennai). The antioxidant activity of AgNPs was expressed as IC_50_, the concentration (μg/mL) of AgNPs that inhibits the formation of DPPH radicals by 50%. The scavenging activity of AgNPs was compared with the standard ascorbic acid (ASA) and *S. acmella* leaf extract (SAAE), and each test was performed in triplicate. The scavenging activity was then estimated based on the percentage of DPPH radicals scavenged using the formula
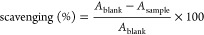
where *A*_blank_ is the absorbance of the control (solution containing all of the reagents except AgNPs) and *A*_sample_ is the absorbance of the solution containing AgNPs.

#### 2.6.2 ABTS Radical Scavenging Activity

The scavenging activity of AgNPs against ABTS was determined according to the method of Re et al.^31^ Briefly, a stock solution was prepared by mixing 5 mL each of 7 mM ABTS and 2.4 mM potassium persulfate. The stock solution was then incubated for 12 h at room temperature in the dark to yield a dark-colored solution that contains ABTS**˙**^**+**^ radicals. A fresh working solution was prepared before each assay by diluting the stock solution with 50% methanol (v/v) having an initial absorbance of 0.70 (±0.02) at 745 nm. The ABTS**˙**^**+**^ radical scavenging activity was then determined by mixing 150 μL of AgNPs (5–40 μg/mL) with 1.5 mL of ABTS working solution. The decrease in absorbance was measured immediately at 745 nm. The scavenging activity of AgNPs was compared with the standard ascorbic acid (ASA) and *S. acmella* leaf extract, and each test was performed in triplicate. The scavenging activity of AgNPs was then calculated using the formula
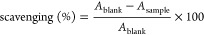
where *A*_blank_ is the absorbance of the control (solution containing all of the reagents except AgNPs) and *A*_sample_ is the absorbance of the solution containing AgNPs.

#### 2.6.3 Superoxide Radical Scavenging Activity

Superoxide scavenging activity of AgNPs was determined by the nitroblue tetrazolium (NBT) reduction method^32^ with minor modifications. Briefly, the reaction mixture was prepared using 0.5 mL of NBT (1 mM NBT in 100 mM phosphate buffer), 0.5 mL of NADH solution (1 mM NADH in 100 mM phosphate buffer), and 0.1 mL of different concentration (1–25 μg/mL) of AgNPs. Then, 100 μL of PMS solution (60 μM PMS in 100 mM phosphate buffer) was added to the mixture to initiate the reaction. The samples were then incubated for 15 min under visible light followed by a measurement of absorbance at 530 nm. The superoxide radical scavenging activity of AgNPs was then calculated using the following formula
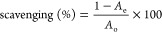
where *A*_o_ is the absorbance without the sample and *A*_e_ is the absorbance with the sample.

#### 2.6.4 Reducing Power

The reducing power of AgNPs was estimated using the method describe earlier^33^ with minor modifications. Briefly, 2.5 mL each of 0.1% potassium ferricyanide solution and 0.2 M phosphate buffer (pH 6.6) were mixed with different concentrations (10–500 μg/mL) of AgNPs. The mixture was incubated for 20 min at 50 °C followed by the addition of 10% TCA (2.5 mL). After centrifugation of the mixture at 3000 rpm for 10 min, an equal volume of distilled H_2_O was added to the supernatant followed by 0.5 mL of 1% ferric chloride solution. The absorbance of the mixture was measured at 700 nm. The increase in absorbance indicated an increase in reducing the power of AgNPs.

### 2.7 *Ex Vivo* Antioxidant Assay

#### 2.7.1 Antihemolytic Activity

The inhibitory effect of AgNPs against mice erythrocyte hemolysis was measured^34^ to determine its antioxidative potential. Blood was collected from healthy adult Swiss albino mice by heart puncture in a heparinized tube. Erythrocyte hemolysis was induced with hydrogen peroxide (H_2_O_2_), which serves as a free-radical initiator. A mixture was prepared by adding 0.25 mL of 5% (v/v) suspension of RBC in phosphate-buffered saline (PBS), 0.2 mL of different concentration (0.1–0.5 mg/mL) of AgNPs, and 50 μL of H_2_O_2_ (1 mol/L). The reaction mixture was gently mixed while being incubated at 37 °C for 3 h. After dilution with 2 mL of PBS, the reaction mixture was centrifuged at 2000 rpm for 10 min. The absorbance of the supernatant was measured at 540 nm. The inhibition rate of erythrocyte hemolysis was then calculated
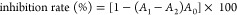
where *A*_0_ is the absorbance of the control, *A*_1_ is the absorbance of the solution containing AgNPs, and *A*_2_ is the absorbance without RBC.

#### 2.7.2 Inhibition of Lipid Peroxidation

Lipid peroxidation inhibitory action of AgNPs was measured according to the method of Gill et al.^35^ using mice liver. The liver was excised from Swiss albino mice, and 1% of liver homogenate was prepared in PBS. The liver homogenate was then centrifuged at 3000 rpm at 4 °C for 10 min, and the supernatant (0.5 mL) was mixed with different concentrations (0.05–0.2 mg/mL) of AgNPs, 0.25 mL each of 25 mmol/L FeCl_2_ and H_2_O_2_, and incubated at 37 °C for 1 h. Absorbance was measured at 535 nm, and the rate of inhibition of lipid peroxidation was calculated using the formula
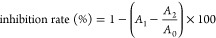
where *A*_0_ is the absorbance of the control, *A*_1_ is the absorbance of the solution containing AgNPs, and *A*_2_ is the absorbance without liver homogenate.

### 2.8 Animals and Tumor Model

All experimental procedures involving animal care and handling were carried out in accordance with the guidance for caring and using of Laboratory Animals (National Institutes of Health) and approved by the Mizoram University Institutional Animal Ethical Committee (MZU/IAEC/2022-23/03). The adult Swiss albino mice weighing 25–30 g were selected and maintained under controlled temperature (25 ± 2 °C) and photoperiod of 12/12 h light/dark cycles (Frontier Euro Digital Timer, Taiwan) at the Animal Care Facility, Department of Zoology, Mizoram University, India. All animals were provided sawdust as bedding and had access to standard food pellets and water *ad libitum*. Dalton’s lymphoma ascites (DLA) tumor has been maintained in 10–12 weeks old mice by serial intraperitoneal (i.p.) transplantation of 1 × 10^6^ viable tumor cells per animal (in 0.25 mL phosphate-buffered saline (PBS), pH 7.4) under aseptic conditions.

### 2.9 Experimental Design

All animals, except the control group, were injected (i.p.) with DLA cells on day 0. The AgNP treatment was carried out for 7 consecutive days. Animals were randomly divided into seven groups consisting of six individuals each (*n* = 6) as follows:

Group I (control group): mice were injected (i.p.) with 0.5 mL of normal saline on day 1.

Group II (DLA group): mice were injected (i.p.) with 0.5 mL of normal saline on day 1 followed by 0.5 mL of distilled water (vehicle) by oral gavage daily.

Group III (DOX group): mice were injected (i.p.) with doxorubicin (20 mg/kg b.wt) on day 1 followed by 0.5 mL of distilled water by oral gavage daily.

Group IV, V, VI (DOX + AgNP groups): mice were injected (i.p.) with doxorubicin (20 mg/kg b.wt) on day 1 followed by different dosages of AgNPs (25, 50, and 100 mg/kg b.wt) by oral gavage daily.

Group VII (AgNP group): mice were injected (i.p.) with 0.5 mL of normal saline on day 1 followed by AgNPs (50 mg/kg b.wt) by oral gavage daily.

### 2.10 Preparation of Tissue Homogenates for Biochemical Assays

After 7 days of treatment, the animals were sacrificed and the liver and heart were immediately excised. Tissue homogenate (5%, w/v) was prepared with ice-cold buffer (5 mM EDTA, 150 mM NaCl, pH 7.4). The homogenates were centrifuged at 13,000 rpm for 30 min at 4 °C, and the supernatants were stored at −80 °C in aliquots until used for biochemical assays.

### 2.11 Estimations of Serum Aspartate Amino-Transferase (AST), Alanine Amino-Transferase (ALT), and Lactate Dehydrogenase (LDH)

Blood was collected by heart puncture using a heparin-coated syringe and centrifuged at 2000 rpm for 15 min at 4 °C. The serum was assayed for AST (EC 2.6.1.1), ALT (EC 2.6.1.2), and LDH (EC 1.1.1.27) according to the manufacturer’s instruction (Transasia Bio-Medicals Ltd., Mumbai, India).

### 2.12 Biochemical Assays

The protein content of the liver and heart was determined using the standard method.^36^

#### 2.12.1 Glutathione (GSH)

Glutathione (GSH) levels were estimated using the method described earlier.^37^ Briefly, 80 μL of 5% tissue homogenate was incubated with a mixture of 20 μL of DTNB (10 mM) and 900 μL of sodium phosphate buffer (0.2 M) for 2 min at room temperature. The absorbance was taken at 412 nm against blank. A mixture devoid of tissue lysates served as blank. The concentration of GSH was calculated from the standard graph and expressed as μmol/mg protein.

#### 2.12.2 Glutathione-*s*-transferase (GST)

The activity of glutathione-*s*-transferase (GST) was determined using the standard method.^38^ Briefly, 50 μL of CDNB (5 mM) was mixed with 850 μL of phosphate buffer (0.1 M; pH 6.5) and incubated at 37 °C for 10 min. To the reaction mixture, 50 μL each of GSH (20 mM) and tissue homogenate were added. A mixture devoid of tissue lysates served as blank. The absorbance was recorded at a 1 min interval for 6 min at 340 nm. GST activity was measured as
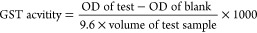
where 9.6 is the molar extinction coefficient for GST.

GST activity was expressed as Unit/mg protein.

#### 2.12.3 Superoxide Dismutase (SOD)

The activity of SOD was estimated by the NBT reduction method^39^ with minor modifications. Briefly, 100 μL each of tissue homogenate and PMS (186 μM) were mixed with 300 μL of NBT (3 mM) and 200 μL of NADH (780 μM). After incubation of the mixture at 30 °C for 90 s, 1 mL of acetic acid and 4 mL of *n*-butanol were added to stop the reaction. A mixture devoid of tissue lysates served as a blank. The absorbance was measured at 560 nm, and the enzyme activity was expressed in the unit (1 unit = 50% inhibition of NBT reduction)/mg protein
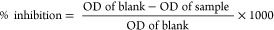


#### 2.12.4 Lipid Peroxidation (LPO) Assay

LPO was measured by the standard method.^40^ Briefly, the tissue homogenate was added to a mixture of 0.8% TBA, 10% TCA, and 0.25 N HCl in a 1:2 ratio. After boiling the mixture for 10 min, it was cooled immediately at room temperature and centrifuged at 12,000 rpm for 10 min. The absorbance of the supernatant was recorded at 535 nm against blank. A mixture devoid of tissue lysates served as blank. The concentration of MDA was calculated using the extinction coefficient of 1.56 × 10^6^ M^**–**1^ cm^**–**1^ and expressed as nmol/mg protein.

### 2.13 Statistical Analysis

Data are expressed as mean ± standard error of the mean. One-way ANOVA followed by Tukey’s test was performed to test significant variations in free-radical scavenging activities, antioxidants status, lipid peroxidation, and serum enzyme activities of treatment groups using SPSS ver.16.0 software (SPSS Inc., Chicago, IL). The IC_50_ was also calculated using GraphPad Prism software ver. 6.0. A “*p*” value of less than 0.05 was considered statistically significant.

## 3 Results and Discussion

### 3.1 Visual Identification and UV–Visible Spectra Analysis

**Figure 1:**
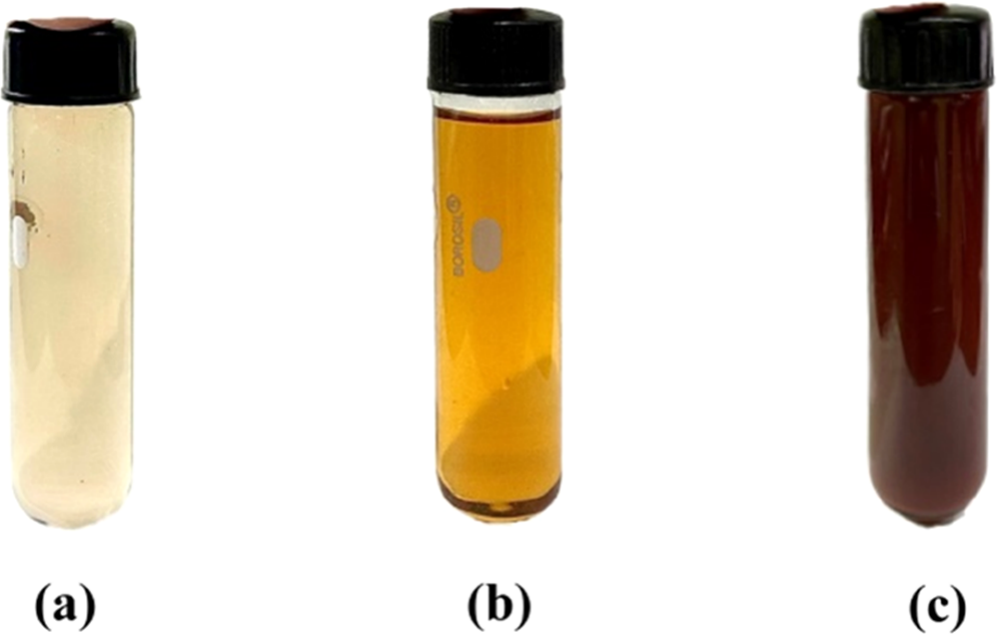
Visual identification of biosynthesized AgNPs as recorded at different time intervals: (a) initial, (b) 2 h, and (c) 4 h. The formation of a reddish-brown color revealed the formation of AgNPs in the reaction mixture.

**Figure 2:**
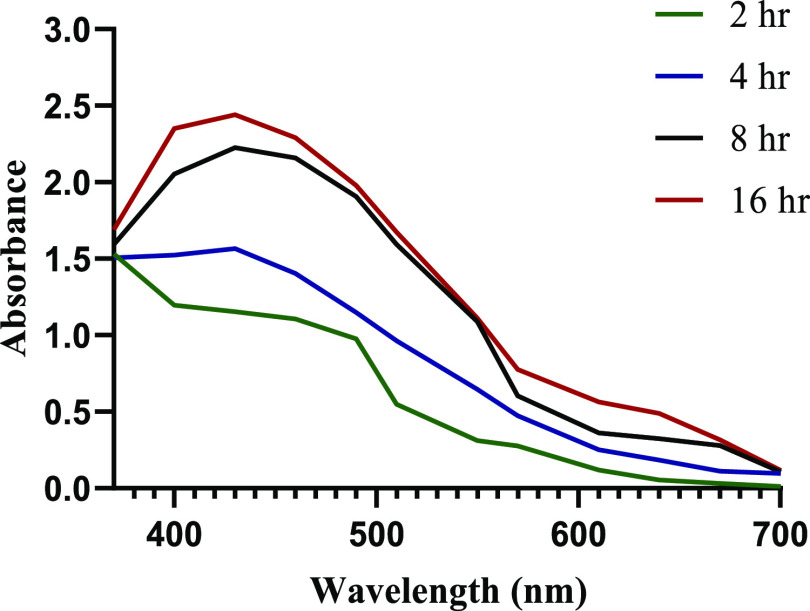
UV–vis absorption spectra of biosynthesized AgNPs using *S. acmella* leaf extract at different time intervals.

Prior to UV–vis spectral analysis, the formation of AgNPs was identified through visual observation of the change in color from pale yellowish-brown to a reddish-dark brown color after 30 min of stirring the mixture (Figure 1). This color change was due to the reduction of Ag^+^ to Ag^0^ (metallic silver) by the bioactive ingredient of the *S. acmella* leaf extract and the excitation of surface plasmon resonance (SPR) with the AgNPs. UV–vis absorbance of the reaction mixture was then recorded at different time intervals. The steady increase in intensity of SPR suggested a gradual increase in the yield of AgNPs with the increase in time. The SPR of the nanoparticles produced a peak centered at 430 nm, indicating the reduction of AgNO_3_ into AgNPs (Figure 2).

### 3.2 FT-IR Spectrum

**Figure 3:**
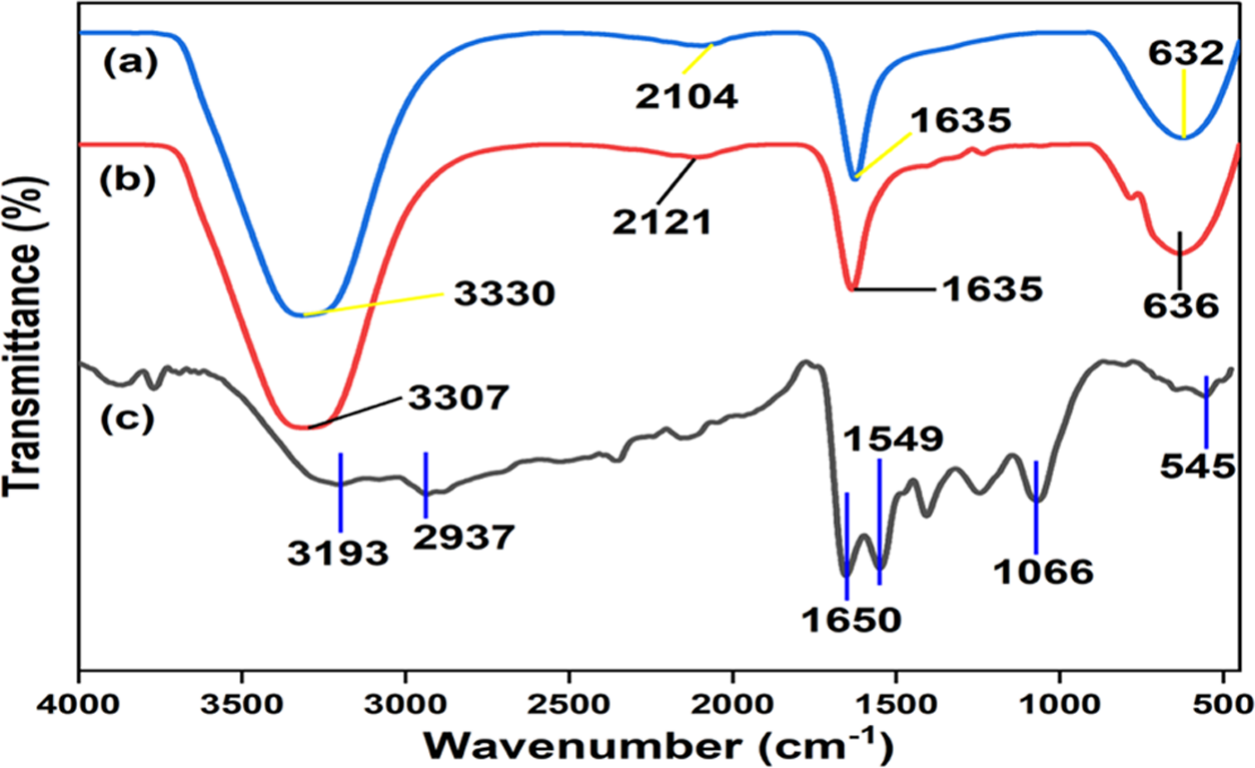
FT-IR analysis of (a) silver nitrate, (b) *S. acmella* leaf extract (SAAE), and (c) biosynthesized AgNPs.

FT-IR spectroscopy was used to investigate the presence of bioactive compounds of *S. acmella* leaf extract in the synthesized AgNPs, which may act as an efficient capping agent and stabilization factors. In the FT-IR spectrum of *S. acmella* leaf extract and its biosynthesized AgNPs (Figure 3), the FT-IR peak observed at 3193 cm^–1^ corresponds to strong stretching vibrations of hydroxyl and amino groups of alcohols and phenolic compounds. The peak at 2937 and 1549 cm^–1^ can be assigned to the C–H-asym. stretching vibration and −CH_3_-sym. stretching vibration, respectively, which indicates the presence of aromatic and carbonyl groups of the protein and metabolites present in the *S. acmella* leaf extract, which is probably involved in the reduction of Ag^+^ to Ag^0.^^41^ The strong peak at 1650 cm^–1^ can be assigned to the stretching of carbonyl groups (C=O), which indicates the presence of compounds like flavonoids and terpenoids^42^ that are responsible for the efficient capping and stabilization of biosynthesized AgNPs. The peak at 1066 cm^–1^ was due to the C–O stretching vibration, whereas the peak at 545 cm^–1^ corresponds to the C–Cl stretching in the alkyl group. The IR spectrum of the *S. acmella* leaf extract exhibited a strong peak at 1635 cm^–1^, which corresponds to the C–O of the amide I protein stretching mode. This peak shifted to 1650 cm^–1^ in the IR spectrum of AgNPs, suggesting the possible involvement of the aforementioned groups in AgNP synthesis by binding the proteins to Ag^+^ through free amine groups or carboxylate ions and indicating the presence of residual *S. acmella* leaf extract in the sample as a capping agent to the AgNPs.

### 3.3 SEM and EDS Analyses

**Figure 4:**
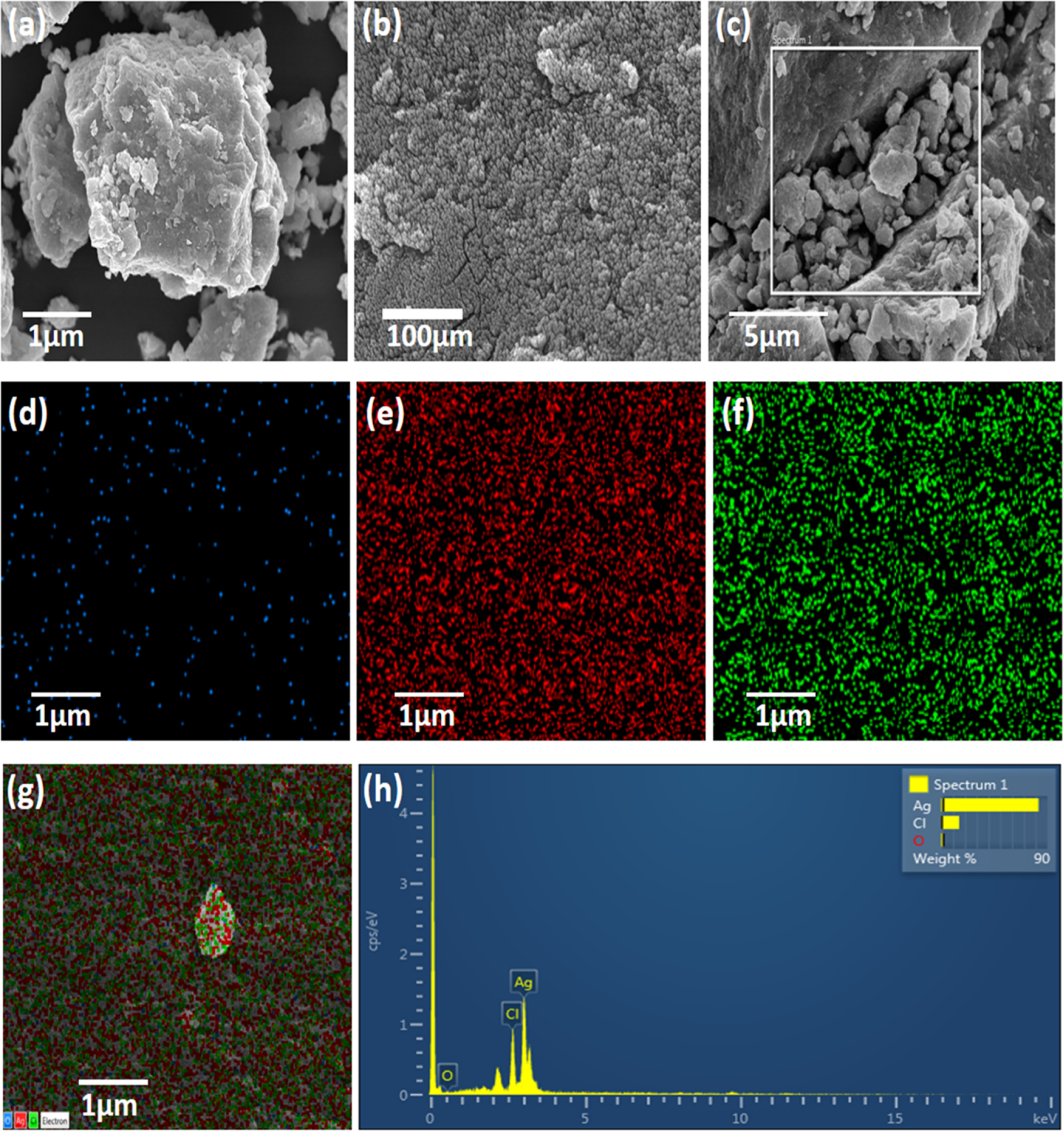
(a–c) SEM micrographs of biosynthesized AgNPs. Elemental mapping of oxygen (d), silver (e), and chlorine (f), and all three elements (g) for the inset region, and (h) EDS data of the area in the white box in panel (c). Scale bars: (a) 1 μm, (b) 100 μm, (c) 5 μm, and (d–g and inset) 1 μm.

Scanning electron microscope (SEM) analysis confirmed the spherical shape of AgNPs in different magnificent images (Figure 4a–c). The occurrence of the elemental silver was indicated by the energy dispersive X-ray spectroscopy (EDS) analysis (Figure 4c,h), which confirmed the reduction of silver ions to silver elements in the reaction mixture. The EDS spectrum also illustrated the presence of strong metallic Ag signals and confirmed the elemental constituents of silver (82.2%), chlorine (15.05%), and oxygen (2.68%) (Figure 4d–h). The strong sharp signal observed for silver is a clear indication of the absorption of the crystalline nature of biosynthesized AgNPs.^44^

### 3.4 TEM Analysis

**Figure 5:**
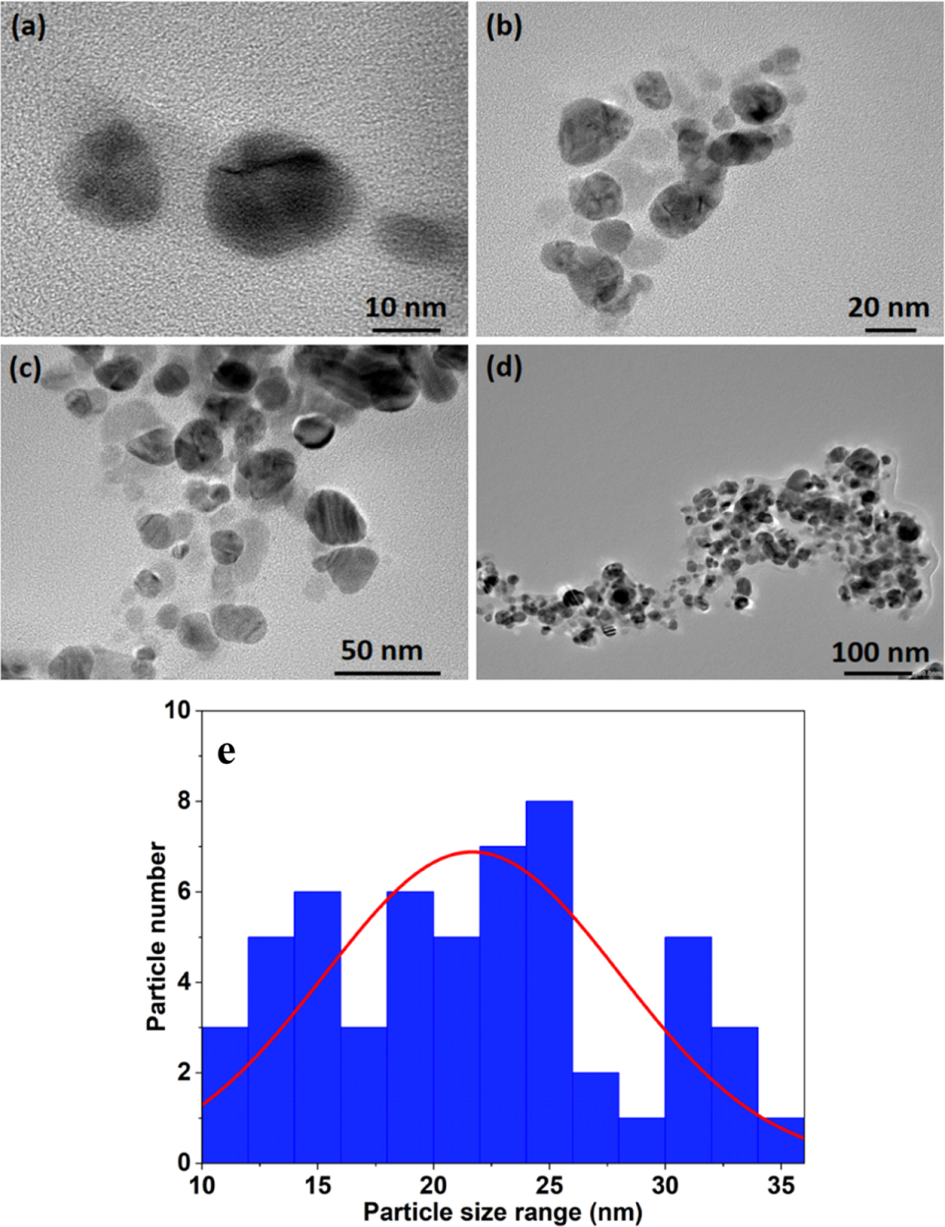
(a–d) TEM micrograph showing the size of biosynthesized AgNPs. Scale bars: (a) 10 nm, (b) 20 nm, (c) 50 nm, and (d) 100 nm. (e) Histogram of the TEM image (100 nm).

TEM analysis elucidates the shape and size of the biosynthesized AgNPs. The TEM images (Figure 5a–d) showed that the biosynthesized AgNPs are polydisperse and are predominantly spherical and oval in shape with particle sizes ranging from 10 to 35 nm (Figure 5e). Hawar et al.^3^ and Khane et al.^4^ have reported the synthesis of spherical AgNPs with an average size of 22–36 and 10–28 nm using aqueous extracts of *Alhagi graecorum* and *Citrus limon*, respectively. Figure 5b shows the biomolecular coating on the surface layer of AgNPs, which is responsible for the enhanced stability of AgNPs.^43^

### 3.5 X-ray Diffraction Analysis

**Figure 6:**
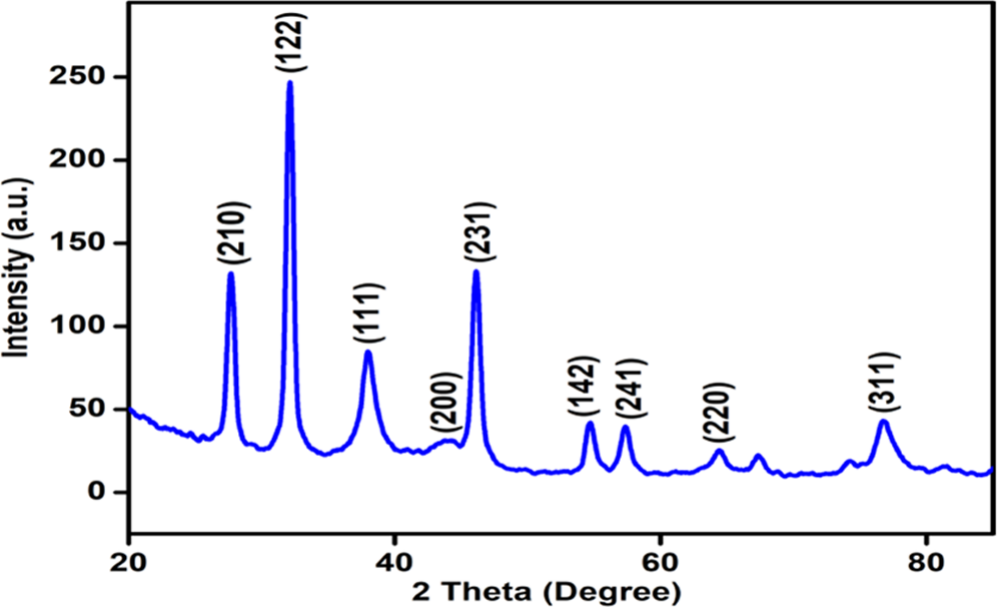
XRD pattern of biosynthesized AgNPs using the *S. acmella* leaf extract.

X-ray diffraction pattern of the biosynthesized AgNPs is shown in Figure 6. At 2θ, values of 27.81, 32.16, 38.12, 44.3, 46.21, 54.83, 57.39, 64.42, and 77.45°, a number of Bragg reflection is observed corresponding to (210), (122), (111), (200), (231), (142), (241), (220), and (311) planes of pure silver based on the face-centered cubic structure (JCPDS file No. 04-0783), indicating the formation of AgNPs. From X-ray diffraction (XRD) results, it is observed that the AgNPs synthesized by *S. acmella* leaf extract are face-centered cubic (fcc) and crystalline in nature. The full width at half-maximum (FWHM) data was used with Scherrer’s formula to determine the mean particle size. Scherrer’s equation is given as *d* = 0.9λ/β cosθ, where *d* is the mean diameter of nanoparticles, λ is the wavelength of the X-ray radiation source, and β is the angular FWHM of the XRD peak at the diffraction angle θ. The crystalline size of the biosynthesized AgNPs as estimated from the FWHM of the peak (111) using Scherrer’s formula was found to be 6.702 nm.

### 3.6 *In Vitro* Antioxidant Assays

**Figure 7:**
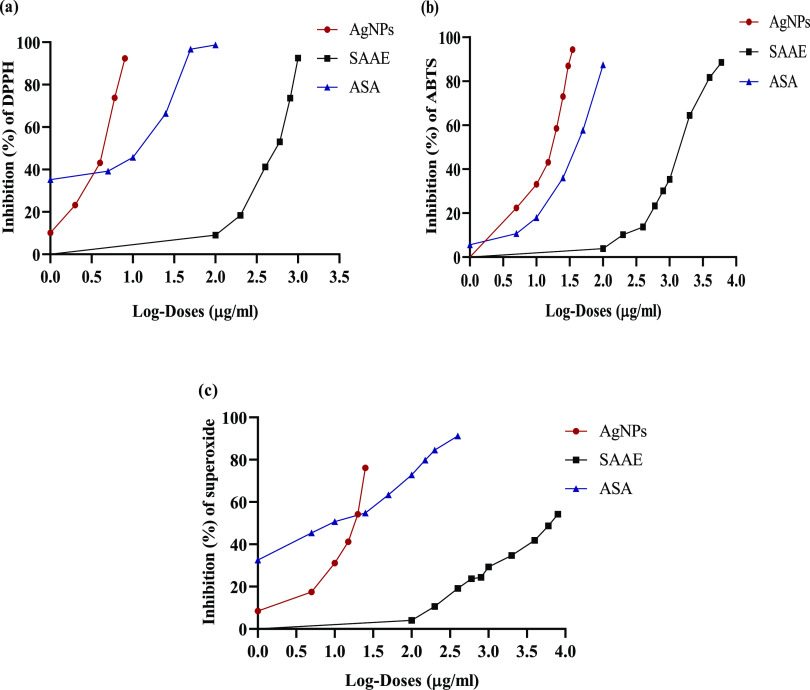
Plots of log-doses of AgNPs, SAAE, and ASA against (a) DPPH, (b) ABTS, and (c) O_2_^•**–**^ inhibition (%) for the calculation of IC_50_. AgNPs: biosynthesized AgNPs using *S. acmella* leaf extract; SAAE: *S. acmella* leaf extract; ASA: ascorbic acid (standard).

**Figure 8:**
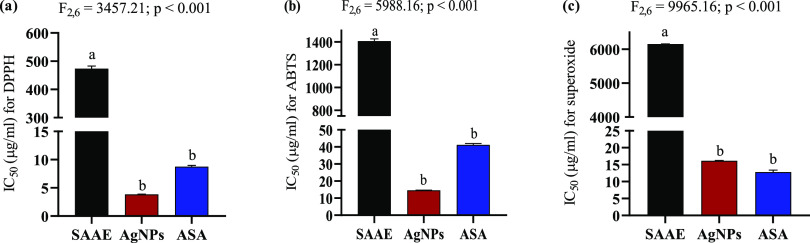
IC_50_ (μg/mL) of AgNPs, SAAE, and ASA for (a) DPPH, (b) ABTS, and (c) O_2_^•**–**^. AgNPs: biosynthesized AgNPs using *S. acmella* leaf extract; SAAE: *S. acmella* leaf extract; ASA: ascorbic acid (standard). Values are expressed as mean ± SEM, *n* = 3. Different letters indicate significant variation.

**Figure 9:**
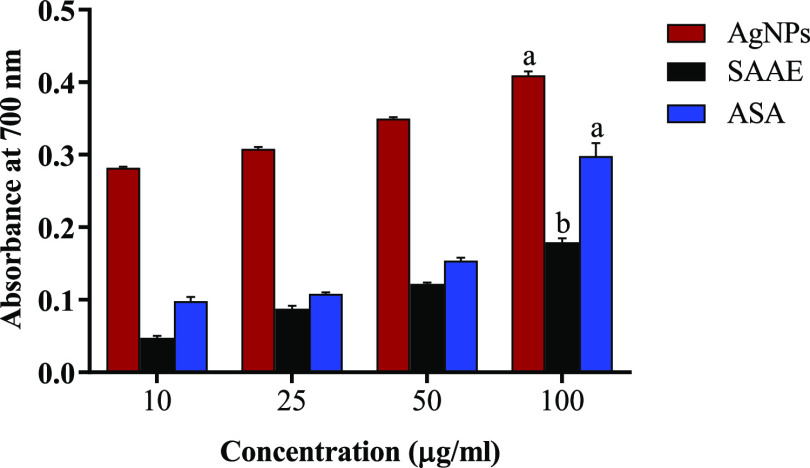
Reducing power of AgNPs, SAAE, and ASA at different concentrations. AgNPs: biosynthesized AgNPs using *S. acmella* leaf extract; SAAE: *S. acmella* leaf extract; ASA: ascorbic acid (standard). Values are expressed as mean ± SEM, *n* = 3. Different letters indicate significant variation.

*In vitro* antioxidant assays using DPPH, ABTS**˙**^**+**^, and O_2_^•**–**^ revealed the antioxidative potential of AgNPs. The free-radical scavenging activities of AgNPs increased in a concentration-dependent manner. Log-doses of AgNPs, SAAE, and ASA were plotted against inhibition (%) of DPPH, ABTS**˙**^**+**^, and O_2_^•**–**^ radicals for the calculation of IC_50_ (Figure 7a–c). The scavenging activities of AgNPs against DPPH (IC_50_: 3.85 ± 0.04 μg/mL), ABTS**˙**^**+**^(IC_50_: 14.62 ± 0.10 μg/mL), and O_2_^•**–**^ (IC_50_: 16.13 ± 0.11 μg/mL) were found to be significantly higher than that of SAAE (IC_50_: 474.0 ± 8.80 μg/mL for DPPH; 1409.33 ± 17.8 μg/mL for ABTṠ^+^; 6156.33 ± 15.23 μg/mL for O_2_^•**–**^). Despite the nonsignificant variations, AgNPs showed better scavenging activities against DPPH and ABTS**˙**^**+**^ when compared to the standard ASA. Similarly, no significant variation (*p* > 0.05) was found between AgNPs and the standard ascorbic acid in O_2_^•**–**^ scavenging activities (Figure 8a–c). The reducing power of AgNPs was assessed by measuring their ability to transform ferric (Fe^3+^) into ferrous (Fe^2+^). The reducing activity of AgNPs also increased in a dose-dependent manner. The total reducing power of AgNPs at 100 μg/mL (0.40 ±. 005) was found to be significantly higher than that of SAAE (0.17 ±. 003) and standard ascorbic acid (0.29 ± 0.01) (Figure 9). The high ferric reducing power of AgNPs observed in this study serves as a significant indicator of their potential antioxidant activity. A highly reactive superoxide anion radical, produced as a result of the incomplete metabolism of oxygen, can lead to tissue damage by inducing lipid peroxidation.^45^ Superoxide (O_2_^•**–**^) radical can decompose to form stronger reactive oxygen species (ROS) such as hydroxyl radicals. Thus, scavenging of O_2_^•**–**^ will inhibit the chain of ROS generation, thereby protecting the cells from oxidative damage.

In the present study, AgNPs could effectively reduce the purple radical DPPH to the nonradical yellow-colored DPPH-H, and ABTS^**•+**^ to ABTS demonstrating the potent antioxidant nature of biosynthesized AgNPs. The green synthesis of AgNPs is largely based on the keto–enol conversion of polyphenolic compounds, which shows strong antioxidant and radical scavenging activities.^46^*S. acmella* has been reported to contain a high amount of polyphenolic compounds.^47^ The primary contributor to the antioxidant properties of plants and plant-derived compounds is the presence of phenolic compounds that have conjugated ring structures and hydroxyl groups that enable them to catalyze the scavenging or stabilization of free radicals involved in oxidative processes *via* hydrogenation or conjugation with oxidizing molecules.^48^ Gold, silver, and selenium nanoparticles have also been shown to effectively alleviate oxidative stress due to their redox-active radical scavenging properties.^43,49^ The free-radical scavenging activities of biosynthesized AgNPs against DPPH, ABTS**˙**^**+**^, and O_2_^•**–**^ could be attributed to bioactive constituents of *S. acmella* leaf extract that adhered to the spherical shape nanoparticles. The bioactive compounds originated from *S. acmella* leaf extract along with the silver ions and may serve as antioxidants *via* transferring of a single electron and hydrogen atom.^50^ The higher scavenging activities of biosynthesized AgNPs than the *S. acmella* leaf extract could be due to the simultaneous action of phenolic compounds as antioxidant agents and silver ions as a catalyst.^51^ The present findings are in line with numerous studies that have reported the free-radical scavenging activities of biosynthesized AgNPs.^4,5,52^

### 3.7 *Ex Vivo* Antioxidant Assay

**Figure 10:**
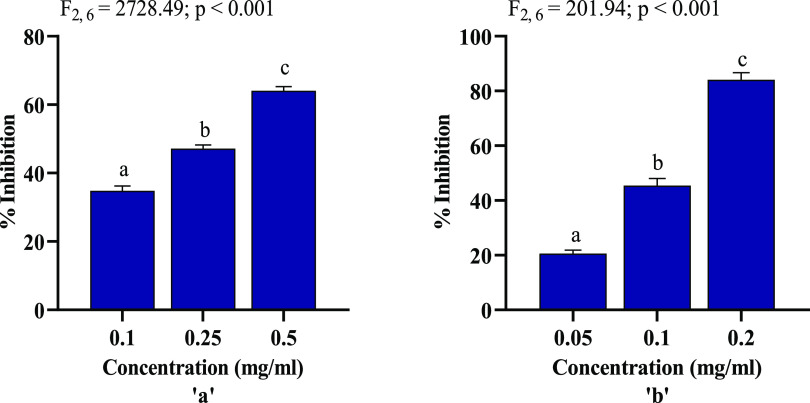
(a) Antihemolytic and (b) lipid peroxidation inhibitory activities of biosynthesized AgNPs. Values are expressed as mean ± SEM, *n* = 3. Different letters indicate significant variation.

The cellular membrane is one of the major targets of free radicals, and hemolysis occurs due to membrane damage caused by the chain reaction of free radicals on erythrocytes.^53^ Peroxidation of lipid moieties such as polyunsaturated fatty acids by the activities of free radicals can lead to membrane damage.^54^ Lipid peroxidation in liver cells of mice and hemolysis was induced using H_2_O_2_, and the inhibitory potential of AgNPs was studied. Both the antihemolytic activity and lipid peroxidation inhibitory effect of AgNPs occur in a concentration-dependent manner (Figure 10a,b). Inhibitory activity of AgNPs against mice erythrocyte hemolysis at a dose of 0.5 mg/mL was 64.07%, indicating the potent antihemolytic activity of biosynthesized AgNPs. The highest inhibitory effect of AgNPs against lipid peroxidation in mice liver homogenate was recorded at 0.2 mg/mL with an inhibition rate of 84.2%. Certain phenolic compounds have been reported to partition cell membrane, hindering free-radical diffusion and consequently reducing the kinetics of free-radical interactions.^55^ Flavonoids have also been reported to inhibit lipid peroxidation in the erythrocyte membrane and improved their integrity against lyses by binding to the membrane.^56^ Our findings suggested that biosynthesized AgNPs, due to their unique physicochemical features and high surface-area-to-volume ratio, may allow its interaction with the lipids of the erythrocyte membrane, thereby showing a protective effect against hemolysis.

### 3.8 Serum Enzyme Assays

DOX usage has been linked to higher levels of serum toxicity markers including alanine transaminase (ALT), aspartate transaminase (AST), and lactate dehydrogenase (LDH).^57^ Due to cellular enzyme leakage, damaged liver cells exhibit increased membrane permeability and altered cell transport function, which increase the serum levels of AST and ALT.^58^ The protective effect of AgNPs against DOX-induced toxicity was assessed by determining the serum enzyme activities in different experimental groups (Table 1). Administration of DLA mice with 20 mg/kg b.wt of DOX led to a significant (*p* < 0.01) increase in ALT, AST, and LDH when compared to the normal control group as well as untreated DLA mice. Coadministration of DLA mice with DOX (20 mg/kg b.wt) and AgNPs (50 mg/kg b.wt) results in a significant decrease (*p* < 0.01) in serum enzyme activities when compared to DLA mice receiving DOX only, indicating the protective effects of AgNPs against DOX-induced cardio- and hepatotoxicities. The activity of ALT in DLA mice coadministered with DOX and AgNPs reversed to nearly normal mice.

**Table 1:** Effects of Biosynthesized AgNPs on Serum Enzyme Activities^a^^,^^b^

groups	ALT (U/L)	AST (U/L)	LDH (U/L)
normal control	13.56 ± 0.40^a^	97.13 ± 2.10^a^	427.66 ± 2.20^a^
DLA Control	18.63 ± 0.38^b^	11.33 ± 3.71^b^	580.00 ± 5.77^b^
DLA + DOX	36.00 ± 2.12^c^	159.27 ± 2.24^c^	1010.68 ± 8.80^c^
DLA + DOX + AgNPs_50_	17.46 ± 0.83^a,b^	128.40 ± 3.18^d^	502.15 ± 5.57^d^

a Normal control: Healthy mice without any treatment; DLA control: Dalton’s Lymphoma Ascites (DLA) bearing mice without treatment; DLA + DOX: DLA bearing mice treated with doxorubicin (20 mg/kgb.wt); DLA +DOX + AgNPs_50_: DLA mice treated with 20 mg/kg of doxorubicin followed by *S. acmella* silver nanoparticles at the dose of 50 mg/kg b.wt.

b Values are expressed as Mean ± SEM, *n* = 3. Different letters indicate significant variation.

### 3.9 Effects of AgNPs in Antioxidant Status and Lipid Peroxidation (LPO)

**Figure 11:**
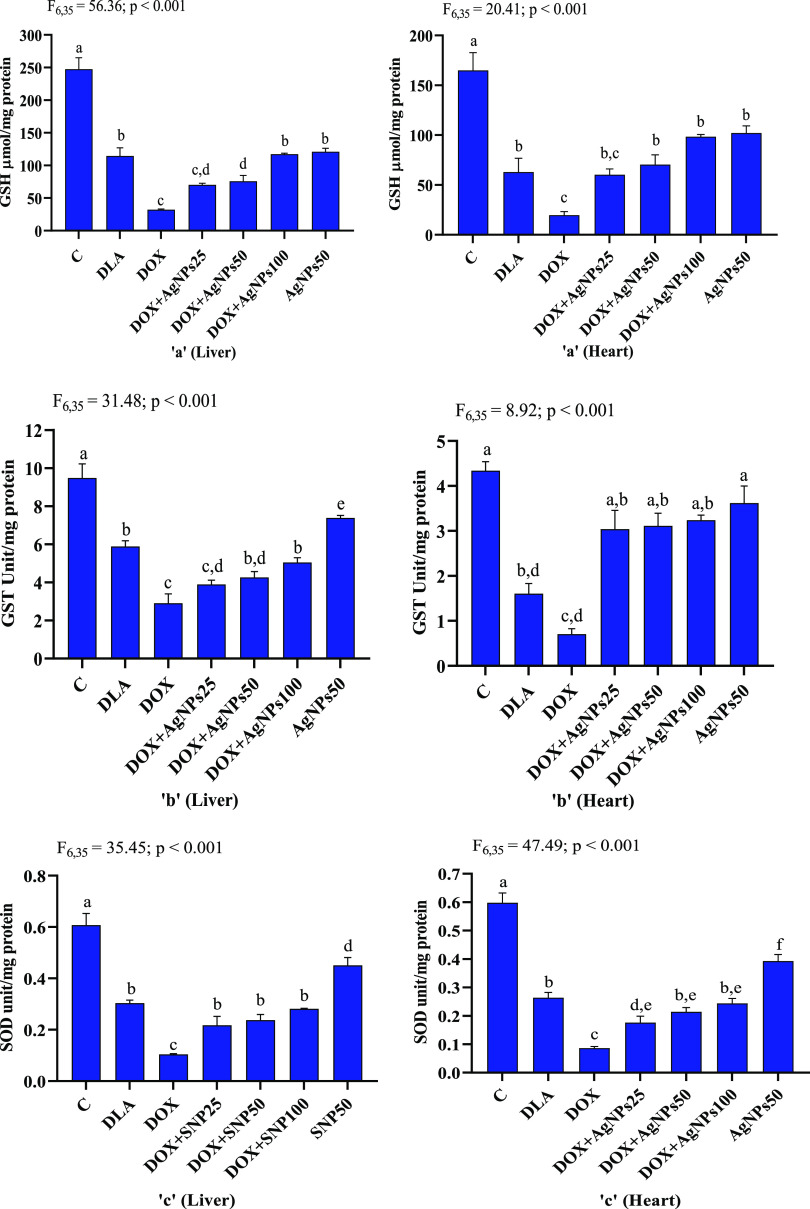
Effects of biosynthesized AgNPs on (a) glutathione level (GSH) (μmol/mg protein), (b) glutathione-*s*-transferase activity (GST) (unit/mg protein), and (c) superoxide dismutase activity (SOD) (unit/mg protein) in the liver and heart of mice. C: normal control; DLA: Dalton’s lymphoma ascites bearing mice without any treatment; DOX: DLA mice treated with 20 mg/kg of doxorubicin; DOX + AgNPs25, DOX + AgNPs50, and DOX + AgNPs100: DLA mice treated with 20 mg/kg of doxorubicin followed by biosynthesized AgNPs at the dose of 25, 50, and 100 mg/kg, respectively. AgNPs50: DLA mice treated with biosynthesized AgNPs (50 mg/kg). Means not sharing the same letter are significantly different.

The long-term use of DOX for cancer treatment has been constrained due to the toxic side effects of the drug that results in organ toxicity. Uses of antioxidants of natural origin have become promising strategies to combat their toxic effects. The deleterious effect of DOX on different organs has been elucidated based on different components, with oxidative stress being considered the most important factor. The oxidative stress caused by a disruption in the antioxidant system can be measured by determining the levels and activities of antioxidants such as glutathione (GSH), glutathione-*s*-transferase (GST), and superoxide dismutase (SOD). In the present study, we investigated the chemopreventive functions of biosynthesized AgNPs using *S. acmella* leaf extract against DOX-induced organ damage in DLA-bearing mice. The antioxidant status was determined in the liver and heart of DLA mice to elucidate the antioxidative potential of AgNPs. DLA mice treated with DOX (20 mg/kg b.wt) showed significantly reduced GSH contents (liver: 3.56-folds; heart: 3.26-folds) and the activities of GST (liver: 2.0-folds; heart: 2.28-folds) and SOD (liver: 3.0-folds; heart: 3.25-folds) in liver and heart when compared to the untreated DLA mice (Figure 11). Coadministration of AgNPs for 7 consecutive days to DOX-treated DLA mice resulted in a significant increase in GSH content and activities of GST and SOD in both the liver and heart of mice. The protective effects of AgNPs against DOX-induced hepato- and cardiotoxicities occurred in a dose-dependent manner. DLA mice treated with AgNPs alone (50 mg/kg bw.t) did not induce any significant change in the GSH contents when compared to untreated DLA mice. However, the treatment of DLA mice with AgNPs alone led to a significant increase in GST and SOD activities as compared to untreated DLA mice, and the increased antioxidant status was comparable to that of the normal control group (Figure 11). Decreased GSH levels following DOX treatment in DLA-bearing mice could be due to excessive utilization of GSH in the liver and heart for scavenging of DOX metabolites. The majority of toxicant covalent binding to hepatic protein has been reported to occur only after GSH depletion.^59^ Improved antioxidant status in DOX-treated DLA-bearing mice after the administration of AgNPs may occur due to maintenance of GSH through neutralization of free radicals by the combined action of bioactive constituents of *S. acmella* leaf extract along with the silver ions. The preventive effects of numerous naturally occurring antioxidants against DOX-induced organ toxicity have been reported earlier.^60^ Phenolic compounds such as oleuropein, sesamol, anthocyanins, curcumin, gingerol, and hydroxytyrosol have been used to reduce DOX-induced toxicity.^61,62^ Similarly, flavonoids, a type of naturally occurring phenolic compounds, such as chrysin, naringenin, kaempferol, avicularin, isorhamnetin, chrysoeriol, hesperidin, apigenin, and baicalein, have been reported to possess protective effects against DOX-induced hepatoxicity, nephrotoxicity, and cardiotoxicity by increasing antioxidant enzymes.^62,63^

**Figure 12:**
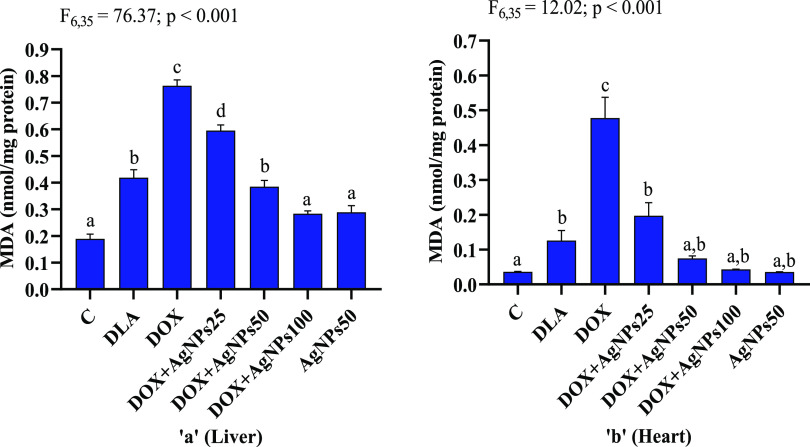
Effects of biosynthesized AgNPs on lipid peroxidation (LPO) expressed in malondialdehyde (MDA) (nmol/mg protein) in (a) liver and (b) heart of DLA mice. C: normal control; DLA: Dalton’s lymphoma ascites bearing mice without any treatment; DOX: DLA mice treated with 20 mg/kg of doxorubicin; DOX + AgNPs25, DOX + AgNPs50, and DOX + AgNPs100: DLA mice treated with 20 mg/kg of doxorubicin followed by biosynthesized AgNPs at the dose of 25, 50, and 100 mg/kg, respectively. AgNPs50: DLA mice treated with biosynthesized AgNPs (50 mg/kg). Means not sharing the same letter are significantly different.

Malondialdehyde (MDA), which is formed during the breakdown of polyunsaturated fatty acids, is a useful index for determining the extent of lipid peroxidation. The most frequently cited evidence to support the involvement of free-radical reactions in toxicity is the detection and measurement of lipid peroxidation.^64^ Administration of DOX to DLA mice resulted in a significant (*p* < 0.001) increase in the MDA levels (liver: 1.83-folds; heart: 3.91-folds) in the liver and heart when compared to that of untreated DLA mice. Coadministration of different doses of AgNPs for 7 consecutive days to DOX-treated DLA mice led to a significant reduction of MDA level (Figure 12). DOX-treated DLA mice that received a higher dose (100 mg/kg bw.t) of AgNPs showed similar MDA levels when compared with the normal control mice. Furthermore, no significant variation (*p* > 0.05) was observed in the level of MDA between DLA mice treated with AgNPs alone (50 mg/kg bw.t) and the normal control group. The increased levels of lipid peroxidation in DOX-treated DLA-bearing mice could be attributed to DOX-induced superoxide anion overproduction and a decrease in detoxifying hydroperoxide. Treatment with biosynthesized AgNPs protects against DOX-induced cellular lipid peroxidation, possibly by inhibiting lipid peroxidation chain reactions in the cytoplasm.

## 4 Conclusions

*S. acmella* contains a wide range of bioactive compounds, such as phenolics, alkyl amides, glycosides, coumarins, triterpenoids, and pyroglutamate with potent anesthetic, antipyretic, analgesic, antifungal, antimalarial, aphrodisiac, vasorelaxant, and immunomodulatory activities. This led us to the synthesis of silver nanoparticles using *S. acmella* leaf extract. The physicochemical properties of biosynthesized AgNPs, such as shape, size, crystalline nature, and the presence of elemental silver, were confirmed by SEM, TEM, EDS, and XRD analyses. The capping of oxidized phenolic compounds and carboxyl protein was responsible for the stability of biosynthesized AgNPs, as confirmed by an FT-IR study. Despite having an effective therapeutic role against various malignant neoplasms, DOX-induced toxic side effects in cancer treatment are well documented. This study found that biosynthesized AgNPs from *S. acmella* leaf extract offer outstanding protections against cardiotoxicity and hepatotoxicity caused by DOX in DLA-bearing mice, possibly by elevating the activities of antioxidants and reduction of lipid peroxidation. Coadministration of biosynthesized AgNPs also reduces the DOX-induced increase in the activities of various serum enzyme markers, confirming its antioxidant potential. Our study also demonstrated that AgNPs synthesized from *S. acmella* leaf extract had great clinical potential for prospective use as anticancer agents by increasing antioxidant levels. However, more research into the precise mechanism of action of biosynthesized AgNPs in protecting against DOX-induced toxicity is needed. Overall, our findings support the use of biosynthesized AgNPs as an effective agent against DOX-mediated toxicity and provide a viable option for increasing doxorubicin’s therapeutic efficacy.

## Notes

The article contains all of the data necessary to understand the conclusions of this investigation.
